# Current Molecular-Targeted Therapies in Melanoma and Their Mechanism of Resistance

**DOI:** 10.3390/cancers18142310

**Published:** 2026-07-17

**Authors:** Rose Bahari, Molly Nguyen, Nayyab Sohail, Stephanie Lopez, Subaranjana Saravanaguru Vasanthi, Jeeya Amin, Dhruv Ramaswami, Georgia Kapetaneas, Riya Karne, Usama Altayeh, Kathryn Joi Rodgers, Aneri Prashant Mehta, Neelu Puri

**Affiliations:** Department of Biomedical Sciences, University of Illinois College of Medicine at Rockford, Rockford, IL 61107, USA; rbahar2@uic.edu (R.B.); mxnguyen@uic.edu (M.N.); nsohai2@uic.edu (N.S.); slope@uic.edu (S.L.); ssara24@uic.edu (S.S.V.); jamin@luc.edu (J.A.); dhruvr@uic.edu (D.R.); georgia6@uic.edu (G.K.); karner@amc.edu (R.K.); ualta@uic.edu (U.A.); krodg7@uic.edu (K.J.R.); apmehta4@wisc.edu (A.P.M.)

**Keywords:** melanoma, cutaneous melanoma, BRAF mutations, NRAS mutations, RAS signaling, MAPK/ERK signaling, MEK inhibition, targeted therapy, immune checkpoint inhibitors, drug resistance, immunotherapy, clinical trials, combination therapy, therapeutic resistance

## Abstract

Melanoma is an aggressive skin cancer that has the potential to metastasize to the lymph nodes, lungs, liver, and brain. Therefore, the prevention and treatment of this condition are essential for achieving lower incidence rates and improving patient outcomes. Traditional treatment methods like surgery, radiation therapy, and chemotherapy have shown limited efficacy in the treatment of metastatic melanoma, and hence new treatment strategies have been developed. These recently developed treatment options include combining targeted therapies with immunotherapies to reduce drug resistance and improve overall effectiveness in preventing melanoma progression. Moreover, *BRAF* mutations are found in approximately 40–50% of cutaneous melanomas, and *NRAS* mutations in 15–25%, making these the two most common oncogenic drivers in the MAPK pathway. While alterations in other genes such as *KRAS* (~1.7%), *HRAS* (~1%), and *MET* (~2–4%) are relatively rare in melanoma, they still remain important to disease biology and are under investigation as potential therapeutic targets. These alterations may contribute to tumor progression, metastasis, and therapeutic resistance, highlighting the importance of continued investigation of targeted strategies in melanoma. This review aims to explore the role of each of these genes in melanoma, discusses their resistance mechanism, and summarizes preclinical and clinical trials involving drug combinations. By integrating current evidence on melanoma-associated genomic alterations with available targeted and immune approaches, this review aims to define molecular and clinical contexts that suggest potential treatment selections for melanoma patients.

## Simple Summary

Drug resistance serves as one of the largest challenges in treating metastatic melanoma. Current studies aim to develop drug combinations using molecularly targeted therapies and immunotherapies to combat drug resistance, which offer patients more effective treatments with fewer side effects, lower recurrence rates, and longer remission periods. These drug combinations delay resistance by targeting multiple pathways involved in tumor growth. This review discusses the mechanism of resistance for genes involved in melanoma, the different combination therapies that have been approved, and the current combinations that are still in preclinical and clinical trials.

## 1. Introduction

According to recent data, in 2022 alone, there were 337,969 new cases of melanoma worldwide and 58,398 melanoma-related deaths [1]. As of 2025, cutaneous melanoma is the fifth most common cancer in the United States [2]. Factors contributing to the development of melanoma typically include consistent sun exposure, family predisposition, melanocytic nevi, dysplastic nevi, and genetic mutations [3].

Many advances have been made in targeted and immunotherapies to improve short-term melanoma patient outcomes. However, despite this, patients may eventually develop resistance to these therapeutic initiatives through various immune and signaling pathways. Resistance may occur by multiple molecular mechanisms, rather than a single genetic change. This includes MAPK reactivation, PI3K/AKT activation, receptor tyrosine kinase (RTK) signaling, and tumor microenvironmental changes.

Among the molecular drivers of melanoma, *BRAF* and *NRAS* represent the dominant oncogenic alterations, while *KRAS*, *HRAS*, and *MET* alterations occur at lower frequencies but still contribute to disease biology in specific molecular subsets [4,5]. Additionally, chronic ultraviolet radiation exposure from the sun can give rise to oncogenic mutations and DNA damage. This damage causes mutations in the Ras family of proto-oncogenes, specifically the *NRAS* proto-oncogene encoding the GTPase protein, leading to the proliferation and differentiation of melanocytes [3,6]. Cases of melanoma arising from early sun exposure or nevi-associated processes are also driven by mutations in the *BRAF*proto-oncogene, specifically the *BRAF *V600E mutation [7]. However, melanoma development is multifactorial, and these associations are not exclusive [3]. The current treatment of malignant melanoma involves targeting these genes with multiple different tyrosine kinase inhibitors (TKIs), as response rates are higher and survival is longer with the combination of TKIs than with monotherapy (Table 1). TKIs have also been used in combination with immunotherapy to mitigate resistance emergence in melanoma cells because resistance develops through multiple pathways. Additionally, combination therapies that simultaneously target these diverse pathways are also becoming increasingly utilized to enhance antitumor immunity (Table 1 and Table 2). Furthermore, there have been many clinical trials that have tested the effectiveness of treatment sequencing and combination therapies, several of which are summarized in Table 2. The clinical trials of KEYNOTE-022, NeoTrio, and DREAMSeq have all served to help develop an understanding regarding combination therapies for patients with the *BRAF* V600E mutation [8]. The KEYNOTE-022 clinical trial tested the effectiveness of PD-L1 immunotherapy in combination with B-Raf/MEK inhibitors [9]. The NeoTrio trial displayed the importance of recognizing the toxicity of certain combination therapies, such as pembrolizumab plus dabrafenib and trametinib [8]. Additionally, the DREAMSeq trials exhibited more positive outcomes for patients treated with nivolumab plus ipilimumab followed by dabrafenib and trametinib. Immune checkpoint inhibitors (ICIs) have also dramatically improved patient outcomes and treatment of malignant melanoma [10]. FDA-approved combination regimens for melanoma are summarized in Table 1, while selected investigational combination strategies are summarized in Table 2. ICIs are currently considered the standard treatment for metastatic melanoma. Monoclonal antibodies targeting CTLA-4 (e.g., ipilimumab) or PD-1 (e.g., nivolumab and pembrolizumab) can trigger the immune system to attack cancer cells [11,12]. Ultimately, the clinical significance of this review lies in its potential to provide more information about treatment and control of metastatic melanoma and an understanding of the genes involved to reduce the effects of drug resistance in melanoma patients.

## 2. B-Raf

An essential part of the MAPK/ERK signaling pathway for controlling cell division and differentiation is the BRAF protein [30]. It is noted that about 35–50% of patients with metastatic melanoma have a *BRAF* V600E mutation, which promotes melanoma progression by activating the MAPK pathway. There are currently several different treatment options that target the *BRAF* gene. B-Raf inhibitors (e.g., dabrafenib and vemurafenib) attach to the mutated B-Raf ATP-binding site, thus blocking the signaling pathway [12]. Unlike conventional chemotherapy, this inhibition promotes apoptosis and slows tumor cell proliferation by targeting cancer cells while reducing the damage to normal cells, thereby reducing adverse effects [31]. Although B-Raf inhibitors have shown improved efficacy, subsequent mutations and activations of neighboring pathways have led to the development of resistance.

### 2.1. MAPK Pathway Reactivation

About 70% of *BRAF*-mutated melanoma resistance cases involve reactivation of the MAPK/ERK pathway via the formation of Raf/c-RAF dimers (B-Raf/B-Rafor B-Raf/c-RAF). This dimer formation is often promoted by mutations in RAS, which increase B-Raf pairing. B-Raf inhibitor drugs only bind to one part of the dimer, leaving the other part drug-free and available for activation. Additionally, the bound portion of the dimer can induce a conformational change in the drug-free portion, thereby activating it and triggering the MEK/ERK pathway. This allows the signaling pathway to remain active despite the presence of an inhibitor, leading to drug resistance [32]. Therefore, combinations of BRAF inhibitors and MEK inhibitors, like trametinib, have been used to inhibit this downstream activation to postpone the development of resistance [15,33].

**Figure 1:**
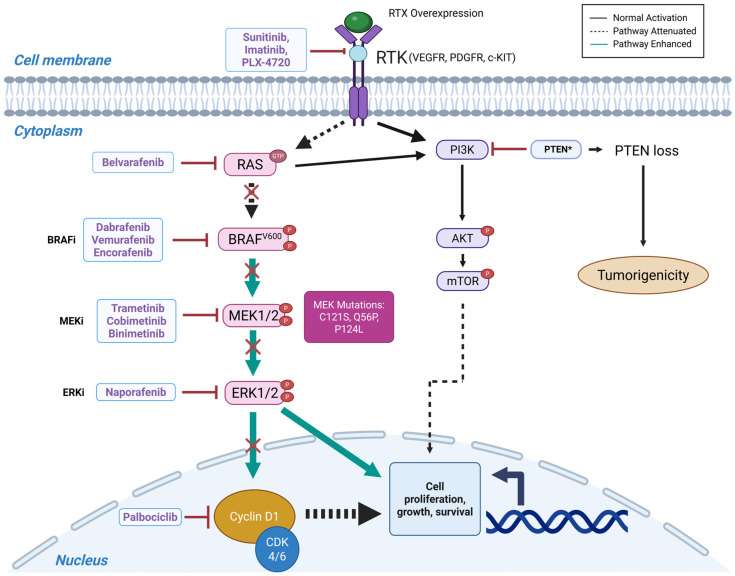
Schematic representation of the PI3K and MAPK signaling pathways in melanoma. The Figure illustrates the major oncogenic drivers that contribute to melanoma cell proliferation and growth. Receptor tyrosine kinases (RTKs) (e.g., VEGFR, PDGFR, and c-KIT) stimulate PI3K and MAPK cascades. *BRAF*V600 drives phosphorylation of MEK1/2 and ERK1/2, thus resulting in transcription of proliferation genes like cyclin D1. This, in turn, activates the cyclin D1 + CDK4/6 complex to result in melanoma cell proliferation. PI3K signaling is suppressed by PTEN; alterations in PTEN* result in loss of function, promoting tumorigenicity. Therapeutic interventions include next-generation RTK inhibitors (e.g., sunitinib and imatinib), BRAF inhibitors (dabrafenib, vemurafenib, encorafenib, and PLX-4720), MEK inhibitors (trametinib, cobimetinib, and binimetinib), and ERK inhibitors (e.g., ulixertinib). Additionally, *MEK1/2* mutations (C121S, Q56P, and P124L) may contribute to acquired resistance to targeted therapies. Inhibition of the cyclin D1–CDK4/6 pathway can be achieved using CDK4/6 inhibitors, such as palbociclib. These targeted therapies can suppress downstream signaling pathways involved in melanoma cell proliferation.

In addition to B-Raf dimerization, acquired resistance to B-Raf inhibitors typically results from mutations that ultimately result in MAPK pathway signaling activation downstream of B-Raf. Secondary mutations in *MEK1* (*MAP2K1*), like P124L, Q56P, C121S, K57N, and F129L, maintain ERK activation while simultaneously reducing sensitivity to MEK inhibition (Figure 1) [34]. Activating point mutations in *MEK2* (MAP2K2), including E207K and Q60I, were also involved in acquired resistance [34]. These mutations additionally reactivate ERK signaling despite continued B-Raf inhibition. Collectively, these secondary mutations represent clinically significant mechanisms of acquired resistance and provide a better understanding of the relation between B-Raf/MEK inhibition and the potential for ERK-targeted therapies.

### 2.2. Alternative Survival Pathways

Tumors may activate other signaling pathways, notably the PI3K/AKT pathway, to circumvent the MAPK/ERK route (Figure 1). The PI3K/AKT/mTOR pathway enables tumor cells to evade apoptosis and becomes overactive due to the increased expression of RTKs, like PDGFRβ and EGFR. This pathway is a critical mechanism contributing to the adaptive resistance to both B-Rafand MEK inhibitors [34]. Loss of *PTEN*, a tumor suppressor gene, has also been implicated in activating the PI3K/AKT/mTOR pathway when B-Raf is inhibited [35]. Hence, tumors can become reliant on alternative pathways for survival and cell growth, leading to resistance mechanisms.

### 2.3. Tumor Microenvironment and Phenotypic Plasticity

Changes in the tumor’s microenvironment can contribute to resistance and further cell growth. Mechanical stress, overcrowding, and upstream signaling can activate the YAP/TAZ pathway. In normal conditions within the Hippo signaling pathway, YAP and TAZ are suppressed through phosphorylation, preventing their localization in the nucleus. However, in BRAFi-resistant melanoma cells, YAP and TAZ proteins translocate into the nucleus due to Hippo pathway inhibition and extracellular matrix remodeling, which increases cell cycle gene expression. YAP/TAZ activation promotes ongoing ERK1/2 signaling via feedback loops or bypassing the blocked BRAF pathway [36]. Similarly, the JNK/c-Jun pathway can be upregulated in response to inflammation or stress. Typically, B-Raf signals ERK to activate c-Jun, which drives cell growth. When the B-Raf pathway is blocked, cells switch to using JNK to activate c-Jun, allowing them to grow regardless of ERK signaling. Therefore, combining B-Raf and JNK inhibitors will be more effective in preventing melanoma progression [34]. Another mechanism of resistance observed in *BRAF*-mutated melanoma cells involves an increase in Wnt-5a protein, which utilizes a β-catenin-independent pathway. In *BRAF*-mutated melanoma, Wnt-5a protein and transcript levels are significantly upregulated through the hyperactive RAF-MEK-ERK MAPK signaling cascade and chronic targeted therapy, promoting therapeutic resistance and tumor survival [37]. This signaling pathway uses the RYK and FZD7 receptors to activate PI3K/AKT, helping cancer cells survive B-Raf inhibition [34]. Other mechanisms of resistance for *BRAF* mutations involve alternative splicing and amplification. Alternative splicing of *BRAF* genes creates shorter B-Raf protein variants that avoid detection by drugs [38]. Together, these mechanisms enable sustained MAPK pathway activation and reduce the long-term efficacy of B-Raf-targeted therapies.

### 2.4. Immune Escape During BRAFi/MEKi Therapy

B-Raf inhibitors alone often lead to resistance and paradoxical MAPK activation in *BRAF* wild-type cells, leading to the development of melanoma. For this reason, B-Raf inhibitors are commonly combined with MEK inhibitors to reduce the likelihood of resistance and downstream MAPK signaling. Although dual therapy with BRAFi/MEKi improves overall survival (OS) compared to monotherapy, resistance typically occurs within 6 to 12 months. Initially, MAPK inhibition increases the body’s immune response by increasing tumor antigen expression and promoting CD8+ T cells [39]. However, adaptive immune resistance has been shown to develop over time due to PD-L1 expression on melanoma cells and sustained PD-1 expression on tumor-infiltrating T cells. This leads to T cell exhaustion and reduced antitumor activity [40]. Immunotherapies—for example, checkpoint inhibitors targeting PD-1 and CTLA-4—are often used as adjuvant treatments to enhance the immune system’s ability to recognize and attack cancer cells.

### 2.5. Dabrafenib, Trametinib, Ipilimumab, or Pembrolizumab

Combination therapy with dabrafenib, trametinib, ipilimumab, and anti-CTLA4 antibodies was previously tested in a Phase I trial (NCT01940809) [10]. However, this combination presented severe side effects, including gastrointestinal toxicity, namely colitis and hepatotoxicity. Due to these significant adverse effects, researchers shifted focus from B-Raf/MEK/anti-CTLA4 therapy to combining B-Raf/MEK inhibitors with immune checkpoint inhibitors such as anti–PD-1 antibodies (e.g., pembrolizumab and spartalizumab) and anti–PD-L1 antibodies. Subsequent trials using anti-PD-1 agents reported response rates up to 85% with improved tolerability; however, direct comparison across trials is limited by differences in study design and patient populations [41].

The Phase II KEYNOTE-022 Part 3 trial further evaluated the efficacy of pembrolizumab in combination with dabrafenib and trametinib compared with placebo plus dabrafenib and trametinib in patients with *BRAF* V600E/K-mutant advanced melanoma. This randomized, double-blind study (*N* = 120) involved patients either receiving triplet therapy or the placebo-controlled doublet therapy of dabrafenib and trametinib. The triple therapy showed significant improvements in the triplet regimen group compared to placebo, with median progression-free survival (PFS) of 16.9 vs. 10.7 months (HR, 0.53; 95% CI, 0.34–0.83) during follow-up studies [9]. Final analysis demonstrated a median OS of 46.3 months versus 26.3 months (HR, 0.60; 95% CI, 0.38–0.95). In addition, there was a longer median duration of response (25.1 vs. 12.1 months) and a 24-month OS rate (63.0% vs. 51.7%) in the triplet regimen [9]. Despite these promising outcomes, triple therapy was associated with a higher rate of grade 3–5 treatment-related adverse events, occurring in 58% of patients, as opposed to only 25% in the doublet group. It is also important to consider that the initial PFS analysis did not meet the trial’s prespecified statistical significance threshold. The preceding hazard ratios were obtained from follow-up studies and were derived from post hoc analyses [9].

The NeoTrio and SECOMBIT are recent Phase II trials that shift focus from solely analyzing the efficacy of combination therapy, to refining the timing and sequencing of dabrafenib, trametinib, and pembrolizumab. The randomized, non-comparative Phase II NeoTrio trial aimed to compare pembrolizumab alone, sequential dabrafenib/trametinib targeted therapies followed by pembrolizumab, and concurrent triple treatment in patients with *BRAF* V600-mutant melanoma. The study found that the concurrent triple treatment yielded the highest response rate (80% vs. 55% with pembrolizumab alone and 50% with sequential therapy). However, very high toxicity and low tolerability rates were found despite having improved pathological responses [8]. Two-year event-free survival was 71% with concurrent triple therapy, 80% with sequential therapy, and 60% with only pembrolizumab [8]. Ultimately, it was determined that the toxicity of triple therapy was too drastic and therefore is not encouraged. Moreover, the SECOMBIT Phase II trial investigated which sequence yields the best long-term survival in metastatic *BRAF*-mutant melanoma, specifically whether to start with targeted therapy or immunotherapy first. The study outcomes showed that starting with immunotherapy and following it with targeted therapy provided the longest survival, especially in patients with specific biomarkers. Patients with low baseline IFNγ and/or *JAK2* mutations responded better to immunotherapy-first (ICI-first) therapy. Together, these two studies highlight the importance of immunotherapy in melanoma treatment and emphasize the need to optimize clinical outcomes by adjusting the timing and sequencing of drugs [42].

In the DREAMseq Phase III Trial, studies were conducted to determine whether the treatment sequence of immunotherapy or targeted therapy first was most effective in patients with treatment-naive *BRAF* V600-mutant metastatic melanoma [10]. Eligible patients were randomly assigned after being stratified by Eastern Cooperative Oncology Group Performance Status (ECOG PS) of 0 or 1 and normal or elevated LDH levels. Additionally, patients may have received adjuvant therapy, but it could not have included adjuvant PD-1/CTLA-4 or B-Raf/MEK inhibitors. In the open-label, randomized Phase III trial study (*N* = 265), patients received either nivolumab/ipilimumab checkpoint inhibitor immunotherapy (Arm A, *N* = 135) or dabrafenib/trametinib targeted therapy (Arm B, *N* = 132) during step 1. To enroll in step 2, patients with RECISTv1.1-documented progressive disease were required to receive either alternative treatment: dabrafenib/trametinib (Arm C) or nivolumab/ipilimumab (Arm D). Ultimately, the independent Data Safety Monitoring Committee stopped the trial early because of a clinically significant endpoint being achieved with initial immunotherapy. This primary endpoint involved a 2-year OS rate, which was higher in those starting on Arm A versus Arm B (71.8% [95% CI, 62.5–79.1] vs. 51.5% [95% CI, 41.7–60.4]; *p* = 0.010) [10]. Upon follow-up studies, there were improved survival benefits with 5-year OS rates being 63.3% versus 33.9%, respectively, and a median PFS of 26.7 months versus 8.5 months (*p* < 0.01) [43]. The incidence of grade ≥3 treatment-related adverse events was compared between treatment arms and within each treatment approach in steps 1 and 2, and the differences were not statistically significant. Subgroup analyses from the 5-year update revealed that there were fewer CNS first progressions (24 vs. 44 patients) and increased time to CNS progression (12.2 vs. 8.4 months; *p* < 0.01). There was also an enhanced response among patients treated with Arm A versus Arm B (76% vs. 24%) [19]. Ultimately, the study established that the combination nivolumab/ipilimumab treatment regimen, administered first, resulted in a 20% absolute improvement in 2-year OS compared to dabrafenib/trametinib. Therefore, the preferred treatment protocol in this patient population is to take a combination of immunotherapy followed by B-Raf/MEK inhibitor therapy.

### 2.6. Vemurafenib, Cobimetinib, Atezolizumab (Anti-PD-L1), or Ipilimumab (Anti-CTLA-4 Antibody)

Another powerful combination involves pairing the B-Raf inhibitor vemurafenib with the MEK inhibitor cobimetinib, along with checkpoint inhibitors like atezolizumab (a PD-L1 inhibitor) or ipilimumab (a CTLA-4 inhibitor). When vemurafenib and atezolizumab were used together in patients with melanoma, extremely high toxicity levels were experienced. In the Phase III IMspire150 trial, researchers were investigating if adding atezolizumab to vemurafenib and cobimetinib combination therapy would improve outcomes in patients with a *BRAF* V600 mutant melanoma [15]. The randomized, double-blinded, and placebo-controlled study (*N* = 514) entailed patients undergoing a 28-day run-in period with vemurafenib and cobimetinib individually before adding atezolizumab (*N* = 256) or placebo (*N* = 258). This approach improved tolerability by reducing side effects. Adding atezolizumab increased median PFS to 15.1 months, compared with 10.6 months in the placebo group. This difference was statistically significant (HR 0.78; 95% CI, 0.63–0.97; *p* = 0.0025) [41]. Nonetheless, independent review did not find the PFS benefit to be statistically significant (16.1 vs. 12.3 months; HR, 0.85; 95% CI, 0.67–1.07; *p* = 0.16), emphasizing the possibility of bias . For the median OS, there was an increased amount in the triplet regimen; however, this was not statistically significant (39.0 vs. 25.8 months; HR, 0.84; 95% CI, 0.66–1.06) [10]. Grade 3–4 adverse events occurred in approximately 79% of patients in the triplet arm, with the most common being elevated blood creatine phosphokinase, lipase, alanine aminotransferase, and aspartate aminotransferase. Exploratory subgroup biomarker analyses demonstrated that there was a PFS benefit from the triplet regimen in patients with an elevated LDH and PD-L1-negative tumors (HR 0.53; 95% CI 0.29–0.95; *p* = 0.032). Meanwhile, there was no benefit seen in the elevated LDH- and PD-L1-positive subgroup (HR 1.16; 95% CI 0.75–1.80) [15]. The regimen was FDA-approved, making it a relatively effective treatment option for *BRAF* V600-mutant advanced melanoma.

### 2.7. Encorafenib, Binimetinib, and Pembrolizumab

More recent clinical studies explore various combination therapies that integrate B-Raf-targeted treatments with immunotherapies. One such study, the IMMU-TARGET trial (NCT02902042), was a single-arm, Phase I/II, open-label, dose-finding study (*N* = 15) with no comparator arm, investigating the combination of encorafenib, binimetinib, and pembrolizumab in patients with previously untreated *BRAF* V600-mutant advanced melanoma. Patients treated with this combination experienced muscle and liver toxicity as indicated by elevated levels of gamma-glutamyl transferase (GGT), aspartate aminotransferase (AST), and creatine phosphokinase (CPK) [17]. After dose adjustment, toxicity levels decreased, and the overall response rate was 64%, with 41% of patients experiencing PFS at 12 months [17].

The promising results of this drug combination prompted further investigation into the use of encorafenib, binimetinib, and pembrolizumab for treating advanced melanoma with the *BRAF* V600E mutation. Researchers are currently conducting the Phase III STARBOARD trial to compare the efficacy of encorafenib, binimetinib, and pembrolizumab against a placebo plus pembrolizumab, and they are in the process of enrolling patients for this study [18]. While many studies are still underway, compelling evidence suggests that incorporating checkpoint inhibitors through immunotherapy alongside B-Raf/MEK-targeted therapy can help slow the development of resistance in melanoma cells and improve overall patient survival rates.

## 3. MEK

Although B-Raf inhibitors have significantly improved outcomes for patients with *BRAF*-mutant melanoma, their long-term effectiveness is limited by acquired resistance. Many resistance mechanisms restore MAPK signaling by reactivating MEK and ERK downstream of B-Raf, allowing melanoma cells to bypass B-Rafinhibition and continue proliferation. Because MEK1 and MEK2 function immediately downstream of B-Raf in the MAPK/ERK signaling cascade, they represent important therapeutic targets for suppressing abnormal pathway activation. Consequently, MEK inhibitors have become a key component of melanoma treatment, particularly when combined with B-Raf inhibitors to achieve more complete pathway inhibition, delay resistance, and improve PFS and OS compared with B-Rafinhibitor monotherapy [44].

MEK inhibitor selectively targets MEK1 and MEK2 kinases that occupy a central position within the MAPK/ERK signaling pathway. In *BRAF*-mutant melanoma, constitutive activation of this pathway drives uncontrolled cell proliferation, survival, and tumor progression. By inhibiting MEK-mediated phosphorylation and activation of ERK, these agents suppress downstream transcriptional programs that promote tumor growth, resulting in cell cycle arrest, reduced proliferation, and increased apoptosis.

Several MEK inhibitors have demonstrated clinical efficacy in advanced melanoma, including trametinib, cobimetinib, and binimetinib [45]. Trametinib is most commonly administered in combination with the B-Raf inhibitor dabrafenib for patients with *BRAF* V600E mutant melanoma. Clinical studies have consistently shown that combined B-Raf and MEK inhibition produces significantly longer PFS and OS than B-Raf inhibitor monotherapy, while reducing paradoxical MAPK pathway activation associated with B-Raf inhibitors alone [12].

Despite their efficacy, MEK inhibitors can also lead to resistance like B-Raf inhibitors [46]. Resistance to MEK inhibitors frequently develops through mechanisms that restore MAPK signaling or activate alternative survival pathways. These include secondary alterations that reactivate ERK signaling, activation of the PI3K/AKT pathway, receptor tyrosine kinase upregulation, and changes within the tumor microenvironment. Overcoming resistance and improving treatment efficacy is possible when MEK inhibitors are used with B-Raf inhibitors or other targeted medicines [47]. More serious adverse effects from these drugs include cardiomyopathy and interstitial lung disease, both of which require constant observation during therapy [48,49].

MEK inhibitors have given patients with melanoma a far better option for treatment. These medicines, which target a key node in the MAPK/ERK pathway, offer a powerful instrument for reducing tumor growth and enhancing patient outcomes [45]. Understanding resistance mechanisms and optimizing combination therapy are the primary areas of ongoing research aimed at enhancing the efficacy of MEK inhibitors.

### 3.1. Trametinib (MEK Inhibitor) Plus Pembrolizumab (PD-1 Inhibitor)

Trametinib and pembrolizumab are paired together because of their similarly synergistic mechanism of action. Trametinib, a MEK inhibitor, stops tumor cell proliferation by targeting the MAPK/ERK pathway. Pembrolizumab is an anti-PD-1 immune checkpoint inhibitor that blocks PD-1 signaling, preventing immune suppression and restoring T cell activation to promote antitumor activity. This combined effect provides better therapeutic outcomes in treating advanced melanoma, as MEK inhibitors enhance the immune system’s ability to recognize tumor cells [9]. They do so by reducing the immunosuppressive signal and sensitizing the cancer cells. Additionally, they play a role in enhancing T-cell entry into tumor cells. Current research on standard-therapy-resistant melanoma cells indicates that blocking MEK can reduce PD-L1 expression, making it more difficult for tumor cells to evade immune attack [9].

This combination therapy has been employed in clinical practice as a last-resort treatment for patients who have seen no improvement in melanoma regression with immune checkpoint inhibitors (ICIs) or targeted drugs, especially those with *BRAF* mutations [20]. Although this combination of therapies has promising outcomes, further investigation is needed to optimize treatment regimens.

### 3.2. Cobimetinib (MEK Inhibitor) Plus Atezolizumab (PD-L1 Inhibitor)

The combination of cobimetinib, a MEK inhibitor, and atezolizumab, a PD-L1 inhibitor, has gained attention as a potential therapeutic strategy for melanoma and colorectal cancer, particularly in patients with mismatch repair-deficient/microsatellite instability-high (dMMR/MSI-H) tumors [50]. As a MEK inhibitor, cobimetinib blocks tumor growth, and atezolizumab helps the immune system attack existing tumor cells. The role of this combination in triple therapy with vemurafenib was evaluated in the Phase III IMspire150 trial. Results from the trial demonstrated that the addition of atezolizumab to cobimetinib and vemurafenib improved PFS, with a median PFS of 15.1 months compared with 10.6 months for cobimetinib plus vemurafenib alone (HR = 0.78; *p* = 0.025) [15]. Additionally, triple therapy yielded mild-to-moderate toxicity levels with a low discontinuation rate, making it a promising therapy regimen.

### 3.3. Binimetinib, Nivolumab, and Ipilimumab

The benefit of adding a MEK inhibitor like binimetinib to a melanoma therapy regimen was reinforced with the SWOG S2000 Phase II trial (NCT04511013), in which binimetinib, nivolumab, and ipilimumab combination therapy was compared to nivolumab and ipilimumab therapy in *BRAF* V600 mutant melanoma patients, including patients with brain metastasis. The PFS for the triple therapy was 6.2 months compared to 1.5 months with doublet therapy (HR 0.47; *p* = 0.04) [19]. Furthermore, combination therapy helped limit melanoma brain metastasis as indicated by the intracranial PFS of 8.7 months compared to 1.5 months (HR 0.39; *p* = 0.01) [19].

MEK inhibitors are an example of a melanoma treatment that targets the mechanisms downstream of BRAF, but there are also treatment therapies being studied that target the Ras isoforms upstream of B-Raf in the MAPK/MEK pathway.

## 4. HRas

Despite the progress with MEK-based combination therapies, rare Ras alterations still present therapeutic challenges. *HRAS*-mutant melanoma is one such uncommon subtype, distinguished by unique biologic behavior [44,51]. *HRAS* mutations are the least common alteration amongst Ras isoforms in melanoma, occurring in approximately 1% of cases [52]. Transcriptional regulation and biological behavior of *HRAS* differ significantly from those of *NRAS* and *KRAS* [53]. In both normal and malignant tissue, HRas is less abundant than NRAS or K-Ras, which correlates with its lower mutation frequency and oncogenic potential in this disease [54]. While *HRAS* mutations are associated with worse outcomes in HNSCC, no significant prognostic association has been established in melanoma [52].

Farnesyltransferase inhibitors (FTIs) were developed to block Ras membrane localization by inhibiting farnesylation, a post-translational modification required for Ras activation [55]. A critical distinction exists among Ras isoforms: Unlike NRAS and K-Ras, HRas is uniquely dependent on farnesylation for membrane localization. NRAS and K-Ras can undergo compensatory geranylgeranylation mediated by geranylgeranyl transferase-I (GGTase-I), allowing them to bypass the effects of farnesyltransferase inhibitors (FTIs) [56]. This explains why FTIs like tipifarnib have shown activity in *HRAS*-mutant HNSCC but were never expected to be effective against *NRAS*- or *KRAS*-driven cancers. In *HRAS*-mutant melanoma, tipifarnib showed no benefit in a Phase II trial, likely because *HRAS*-mutant melanomas frequently harbor co-occurring MAPK and PI3K pathway alterations that confer intrinsic resistance to FTI monotherapy [28]. Other FTIs, including L-778123 and BMS-214662, were ineffective in treating melanoma and were discontinued due to severe toxicities [57,58]. Lonafarnib, combined with sorafenib (multi-kinase inhibitor), showed in vitro activity, but has not advanced to clinical trials in melanoma [59].

ASN007 (ERK1/2 inhibitor) and copanlisib (PI3K inhibitor) have shown preclinical antitumor activity in Ras-mutant models; however, no clinical data supports efficacy in *HRAS*-mutant melanoma [27]. Overall, the rarity of *HRAS* mutations and the consistent failure of targeted approaches in this lineage limit the current therapeutic relevance of HRas in melanoma [52]. However, there are more common Ras isoforms linked to melanoma that are also being studied, like NRAS.

## 5. NRAS

**Figure 2:**
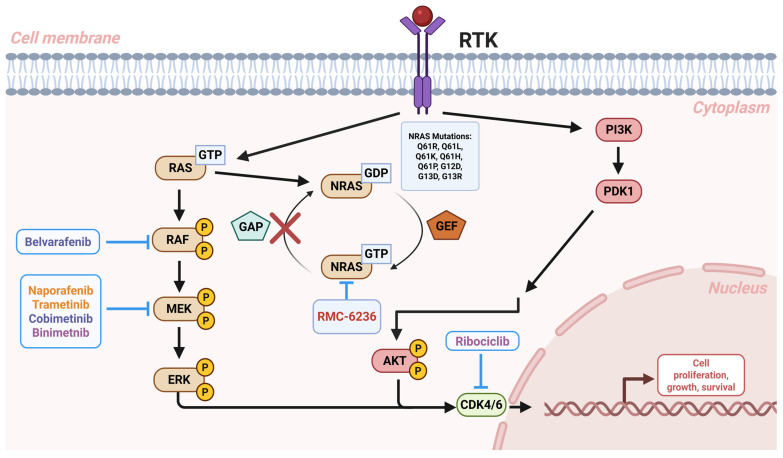
NRAS-mediated signaling pathways and therapeutic targeting strategies in melanoma. Receptor tyrosine kinase (RTK) activation promotes NRAS signaling through receptor dimerization and autophosphorylation, leading to downstream activation of the PI3K/–AKT and MAPK (RAF–MEK–ERK) pathways. NRAS activation is regulated by guanine nucleotide exchange factors (GEFs), which facilitate the exchange of GDP for GTP. Under normal conditions, GTPase-activating proteins (GAPs) promote GTP hydrolysis, thereby inactivating NRAS. However, activating NRAS mutations impair this regulatory mechanism, resulting in sustained NRAS signaling and persistent activation of the MAPK pathway. Dysregulation of the PI3K/AKT and MAPK pathways promotes tumor cell proliferation, growth, and survival. Therapeutic approaches targeting these signaling cascades include RAF inhibitors (belvarafenib and naporafenib), MEK inhibitors (trametinib, cobimetinib, and binimetinib), and CDK4/6 inhibitors (ribociclib).

While *HRAS*-mutant melanoma is rare and has shown limited response to inhibition, NRAS-mutant melanoma is considerably more prevalent and represents a major RAS-driven subtype with significant clinical relevance [44,51]. *NRAS* mutations are the second most common oncogenic driver in cutaneous melanoma, present in approximately 15–20% of cases [60]. They predominantly affect codon 61, while mutations at codons 12 and 13 are rare. Codon 61 mutations impair GTPase activity, preventing NRAS inactivation and resulting in a constitutively GTP-bound active state that promotes persistent downstream signaling through the MAPK and PI3K/AKT pathways [61]. NRAS activation occurs downstream of RTK signaling, where ligand binding induces RTK dimerization and autophosphorylation, leading to activation of guanine nucleotide exchange factors (GEFs) that promote GDP-to-GTP exchange and formation of active NRAS-GTP. Under normal conditions, GTPase-activating proteins (GAPs) facilitate GTP hydrolysis to terminate NRAS signaling; however, oncogenic *NRAS* mutations disrupt this regulatory process, resulting in sustained activation of the MAPK (RAF-MEK-ERK) and PI3-AKT pathways that promote melanoma cell proliferation, survival, and tumor progression (Figure 2). Clinically, *NRAS*-mutant melanomas are associated with aggressive features, including greater tumor thickness, higher mitotic rate, and poorer prognosis compared with *BRAF*-mutant or wild-type melanomas [62]. Unlike *BRAF*-mutant melanoma, no FDA-approved NRAS-specific targeted therapies currently exist [63].

Immunotherapy with anti–PD-1 agents (pembrolizumab or nivolumab) is a first-line systemic therapy for advanced *NRAS*-mutant melanoma, consistent with treatment guidelines for non-*BRAF*-mutant disease. Combination immunotherapy with nivolumab plus ipilimumab is an alternative first-line option that offers improved response rates and longer PFS, though it also carries a greater risk of toxicity and immune-related complications. When immunotherapy is ineffective or not tolerated, second-line options remain limited; dacarbazine chemotherapy provides low response rates and no proven overall survival benefit. In a retrospective study, prior checkpoint inhibitor exposure did not significantly affect dacarbazine response rates, though the overall response rate remained low at approximately 12% [64].

Because NRAS signals through the MAPK pathway, MEK inhibitors have been the most extensively studied targeted therapy approach. However, no MEK inhibitor has received FDA approval for *NRAS*-mutant melanoma, and all combination strategies described below remain investigational. In the Phase III NEMO trial, binimetinib improved progression-free survival compared with dacarbazine (median PFS 2.8 vs. 1.5 months; HR 0.62), though no overall survival benefit was demonstrated [65]. Given the limited durability of MEK inhibitor monotherapy, current trials are evaluating combination strategies that either suppress the MAPK pathway more completely through vertical inhibition (pan-RAF plus MEK) or block parallel escape pathways (CDK4/6 plus MEK, HDACi plus MEK, BCL-xL plus MEK). A Phase Ib/II trial evaluated the combination treatment of binimetinib (MEK inhibitor) and ribociclib (CDK4/6 inhibitor) in patients with locally advanced or metastatic *NRAS*-mutant melanoma. In Phase II, the overall response rate was 19.5%, with a median PFS of 3.7 months and OS of 11.3 months. Patients with simultaneous cell cycle gene mutations had a higher response rate of 32.5% than those without. Common side effects include creatinine phosphokinase elevation, rash, edema, anemia, nausea, diarrhea, and fatigue, with 16.4% of patients experiencing dose-limiting toxicities during the first treatment cycle [21]. Notably, 16.4% of patients experienced dose-limiting toxicities during the first treatment cycle, highlighting the narrow therapeutic window of this combination.

The Nautilus Phase Ib/2 trial examined binimetinib with an oral histone deacetylase inhibitor (bocodepsin) in patients with advanced *NRAS*-mutant melanoma who had already received immunotherapy. This single-arm study reported that 30% of patients responded to treatment, with a median PFS of 7.25 months. No grade 3 or 4 toxicities or high-grade rashes were reported in more than 10% of patients, though the small sample size limits definitive conclusions [22].

In a single-arm Phase Ib study, trametinib (MEK inhibitor) and naporafenib (pan-RAF inhibitor) were evaluated in pretreated patients with *NRAS*-mutant melanoma, the majority of whom had received prior checkpoint inhibitor therapy. At the recommended Phase II dose (*N* = 15), the combination yielded an overall response rate of 46.7% and a median PFS of 5.52 months [23]. The most common treatment-related adverse events were rash (80%), elevated creatine phosphokinase (30%), diarrhea (30%), and nausea (30%); grade 3 or higher toxicities included dermatitis acneiform and maculopapular rash. Based on these results, a Phase III trial is currently evaluating this combination in patients with Ras Q61X-mutant solid tumors [66].

A single-arm Phase Ib trial evaluated belvarafenib (selective RAF dimer inhibitor) and cobimetinib (MEK inhibitor) in patients with *NRAS*-mutant melanoma, including those who had already received checkpoint inhibitor therapy. The study reported a PFS of 7.3 months and an objective response rate of 38.5%. Side effects included acneiform rash, diarrhea, and elevated creatine phosphokinase, but overall the treatment was tolerable [24].

In a single-arm, multicenter Phase II trial (*N* = 95), tunlametinib (MEK inhibitor) was assessed in patients with unresectable stage III/IV *NRAS*-mutant melanoma. The study reported an objective response rate of 35.8%, with a median PFS of 4.2 months and OS of 13.7 months. Patients with previous immunotherapy treatment showed an increased response rate of 40.6% [25]. The most frequent treatment-related adverse events were elevated creatine phosphokinase, diarrhea, facial edema, peripheral edema, and elevated AST; no treatment-related deaths occurred. Tunlametinib received regulatory approval in China in March 2024 for *NRAS*-mutant advanced melanoma after anti-PD-1/PD-L1 failure, but has not been approved in the United States or Europe [67].

A Phase I/II study evaluated navitoclax (BCL-xL inhibitor) and trametinib (MEK inhibitor) in patients with RAS-mutant solid tumors, including *NRAS*-mutant melanoma. The strongest responses were seen in gynecologic cancers (not melanoma-specific), but the combination was tolerable and showed some activity across RAS-mutant solid tumors, though the melanoma-specific subgroup was small and responses were primarily observed in gynecologic cancers [26].

Other early-phase studies are looking at different combinations such as pan-RAF inhibitors with MEK inhibitors, or MEK inhibitors paired with CDK4/6 or histone deacetylase inhibitors [68,69]. However, these approaches are still investigational and not part of standard care.

The limited development of NRAS-targeted therapies in melanoma largely reflects the difficulty of directly inhibiting *NRAS*. However, another Ras isoform, K-Ras, is also being investigated as a therapeutic target for resistant melanoma.

## 6. K-Ras

Another member of the Ras family is *KRAS*, whose mutations are far less common in melanoma than *NRAS*, but remain relevant. *KRAS* is one of the most well-characterized oncogenes with the highest mutation rate among all cancers [44,70]. It is associated with a series of fatal cancers, including, but not limited to, pancreatic ductal adenocarcinoma, colorectal cancer, and non-small cell lung cancer [71]. The protein that *KRAS* encodes is identified as a membrane-bound regulatory protein (G-Protein) that acts by binding to guanosine triphosphate (GTPase) [71]. Regarding melanoma, *KRAS* mutations are rare and only comprise 1.7% of cases, occurring most exclusively in the codon G12 [72].

Although targeted therapies are a promising treatment across many oncogenic drivers, there have still been no effective strategies targeting K-Ras thus far. Consistent with these other challenges, it has been found that patients with *KRAS* mutations have a poor response to current standard therapy options [72]. This can be seen as a significant obstacle to patient care for patients who suffer from the rare *KRAS* mutation variant of melanoma. With less successful treatment outcomes and fewer options to present to patients, one can see how this is a significant area for further research exploration and improvement in patient outcomes.

## 7. c-MET

**Figure 3:**
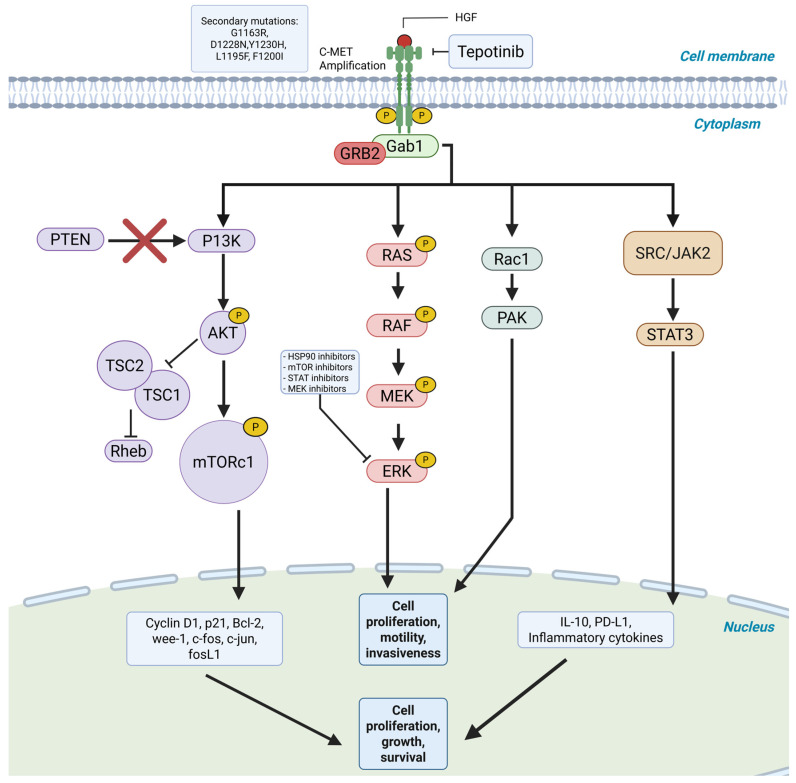
c-MET/HGF signaling cascade and downstream molecular mechanisms promoting tumor progression in melanoma. Binding of hepatocyte growth factor (HGF) to the c-MET receptor tyrosine kinase induces receptor dimerization and autophosphorylation, resulting in activation of multiple downstream signaling cascades, including the MAPK (RAF–MEK–ERK), PI3K–AKT, and Gab1 pathways. HGF-mediated c-MET phosphorylation amplifies MAPK and PI3K–AKT signaling. Activation of the Gab1–GRB2–SOS complex further promotes downstream signaling through the STAT3 and Rac1–PAK pathways. PTEN functions as a negative regulator of PI3K signaling, and loss of PTEN activity results in enhanced activation of PI3K–AKT pathway. Activation of the PI3K–AKT and STAT3 pathways promotes tumor cell proliferation, growth, and survival, whereas MAPK and Rac1–PAK signaling contributes to cellular proliferation, migration, and invasion. c-MET signaling also interacts with and can modulate epidermal growth factor receptor (EGFR)-mediated pathways, contributing to oncogenic signaling and therapeutic resistance.

Unlike the Ras family of oncogenes, c-MET is an RTK that contributes to melanoma progression and resistance by regulating melanoma cell proliferation, metastasis, and angiogenesis. The signaling pathway is activated when c-MET is found bound to its ligand, hepatocyte growth factor (HGF), initiating downstream signaling pathways that promote tumor growth and survival (Figure 3) [73].

Hepatocyte growth factor (HGF)/c-MET signaling is involved in complex cellular programs essential for embryonic development and tissue regeneration, but cancer cells can also use this pathway to support tumor growth and progression [74]. In normal skin cells, dermal fibroblasts are the primary source of HGF, while melanocytes express the c-MET receptor and respond to HGF signaling. This interaction helps protect melanocytes from cell death and promotes their growth and movement. In melanoma, tumor cells can express both HGF and c-MET, allowing continuous activation of this pathway. Increased HGF/c-MET signaling promotes melanoma cell proliferation, survival, migration and invasion, contributing to tumor development and disease progression [74].

Given the significant contribution of c-MET to the development and metastasis of melanoma, targeted therapies addressing this pathway are a promising approach for therapeutic interventions. Rather than one specific pathway, there are several mechanisms that result in resistance to c-MET targeted therapy. *MET* and *KRAS* gene amplification have been identified as potential mediators of resistance to MET kinase inhibitors, as these genes contribute to activation in the MAPK pathway. When HGF/c-MET is activated, it can promote PI3K/AKT downstream signaling, resulting in melanoma cell survival, proliferation, invasion, and migration (Figure 3) [73,74].

Combination targeted therapy has been considered a promising option, targeting specific pathways like c-MET, mTOR, and Wnt, with inhibitors that have the potential to delay the onset of resistance and improve overall patient outcomes. Resistance to c-MET targeted therapies can occur through multiple mechanisms such as *MET* amplification and activation of downstream signaling pathways. *MET* amplification can increase c-MET expression and enhance HGF/c-MET signaling, allowing melanoma cells to maintain tumor-promoting signals despite c-MET inhibition [44]. Additionally, activation of K-Ras can promote continued MAPK pathway signaling, providing an alternative mechanism for melanoma cells to sustain proliferation and survival when c-MET signaling is blocked [44]. Because c-MET signaling interacts with multiple downstream pathways, including MAPK and PI3K/AKT, combination approaches targeting these pathways may help prevent or delay therapeutic resistance (Figure 3) [27]. Several studies using c-MET inhibitors, alone or in combination therapy, for melanoma are currently ongoing. These studies will determine their effectiveness in melanoma patients and their ability to overcome treatment resistance.

Tepotinib, a highly selective oral c-MET inhibitor that was well tolerated, has shown promising efficacy in advanced hepatocellular carcinoma patients with c-MET overexpression. Furthermore, it is currently being investigated for other c-MET-deregulated cancers [75]. Due to its high retention in tumors, tepotinib can block c-MET as well as its downstream pathways and serve as a potent drug for the treatment of several types of cancer [29]. Activation of MET and PI3K/AKT signaling pathways may be blocked by tepotinib treatment. It has been reported that c-MET is overexpressed in melanoma and contributes to its malignant progression, which involves cellular proliferation and cancer formation. Tepotinib has also demonstrated effectiveness within melanoma cells. Specifically, tepotinib has been shown to cause increased apoptosis in the melanoma cell line WM451 through reduced activation of c-MET and inhibition of the PI3K/AKT pathway [29]. However, it is important to note that evidence supporting c-MET inhibition, particularly regarding tepotinib, is derived from preclinical studies and has not been validated in a melanoma-based clinical setting, unlike other forms of cancer. Due to the role of the PI3K/AKT pathway in melanoma progression and cancer cell proliferation, tepotinib-based inhibition of this pathway may serve as a potential therapeutic option for melanoma patients [29,75]. Overall, targeted therapies, immunotherapies, and combination treatment strategies have the potential to improve clinical outcomes in patients with c-MET-altered cancers.

## 8. Discussion

Metastatic melanoma remains one of the most challenging cancers to treat due to its complexity and high therapeutic resistance [76]. Although B-Raf and MEK inhibitors produce high initial response rates in patients with *BRAF*-mutant melanoma, their long-term effectiveness is limited. This is because adaptive resistance mechanisms, including MAPK pathway reactivation, activation of the PI3K/AKT pathways, RTK upregulation, B-Raf splice variants, and alterations within the tumor microenvironment, enable melanoma cells to survive despite targeted inhibition [40]. These findings demonstrate that therapeutic resistance is a complex biological process requiring treatment strategies capable of targeting multiple resistance pathways simultaneously [76,77].

Clinical trials such as KEYNOTE-022 and IMspire150 have demonstrated that combining targeted therapy with immune checkpoint inhibitors can improve PFS, duration of response, and OS compared with targeted therapy alone. By simultaneously suppressing oncogenic MAPK signaling while restoring antitumor immune response through PD-1 or PD-L1 blockades, these combination regimens address both tumor-intrinsic and immune-mediated mechanisms of resistance [9,15]. The improved clinical outcomes observed with triple therapy support the use of combination treatments that target cancer growth while enhancing immune responses to improve long-term control and delay resistance [41]. Although these regimens are associated with increased toxicity, clinical trials have shown that adverse events can be managed through dose modifications, careful patient selection, and optimization of treatment strategies while maintaining clinical benefit [15].

Despite the strides made in this research, several important challenges remain. One major challenge is the identification of reliable predictive biomarkers to guide treatment selection and sequencing [78]. While biomarkers such as PD-L1 expression, lactate dehydrogenase (LDH) levels, circulating tumor DNA, and tumor mutational burden have shown potential value in predicting response and outcomes to immune checkpoint inhibitors, their routine clinical application remains challenging due to limitations in validation [79]. As a result, determining the optimal sequence of targeted therapy and immunotherapy remains uncertain, as treatment decisions depend on the individual’s tumor characteristics, genomic alterations, and other factors that influence therapeutic response [80,81].

Another significant challenge is the emergence of resistance following both targeted therapy and immune checkpoint blockade, including cases in which patients experience primary or acquired resistance [82]. Because these heavily pretreated patients are under-represented in current clinical trials, therapeutic options after treatment failure are limited [83]. Addressing this population will require continued development of comprehensive treatment strategies that incorporate the complex biology of melanoma and improve patient responses for those with limited therapeutic options.

Future directions for this research will likely involve further exploring research regarding precision medicine, in which therapies are tailored to a specific patient’s biology. Proper identification of these actionable molecular alterations within tumors through NGS, IHC, and liquid biopsy allows clinicians to choose the appropriate therapeutic approach. Additionally, because of each individual patient’s unique biology, it is also vital to consider the patient’s biological response to certain drugs and consistently adapt treatment approaches. As a result, an increased emphasis on liquid biopsy tests may provide a less invasive method to track mutations or resistance. Developing biomarkers to identify target areas and determine the most effective drug combination is critical. It is crucial to understand the key genetic alterations that drive the development of these mutant melanomas to effectively target the interactions between tumorigenic cells and their microenvironment. Finally, closing the gap between the population in current trials to reflect the heterogeneity of the real-world population is essential. This includes those patients who are a part of a less observed population or with significant comorbidities.

## 9. Conclusions

The future of metastatic melanoma treatment will depend less on developing more single drugs and more on using molecular testing, predictive biomarkers, and combination therapies that target tumor growth signals and help the immune system fight the cancer. Achieving this goal will require improved biomarkers to help choose the right treatments, new strategies for patients who develop resistance to both targeted therapy and immunotherapy, and clinical studies that include a more diverse group of patients. As resistance mechanisms become better understood, personalized treatment approaches are likely to provide the best chance of achieving durable responses, delaying disease progression, and aiding in long-term survival of patients with advanced melanoma.

## Figures and Tables

**Table 1:** FDA-approved combination regimens for melanoma.

Drug 1 (Target)	Drug 2 (Target)	Drug 3 (Target)	Mutation Context	Study Type	Refs.
***BRAF*** ** V600-Mutant—Targeted Therapy Doublet**					
Dabrafenib (B-Raf)	Trametinib (MEK)	—	*BRAF* V600E/K	FDA-Approved (2014/2015; COMBI-d, COMBI-v)	[13,14]
***BRAF*** ** V600-Mutant-Targeted Therapy + Immunotherapy Triplet**					
Vemurafenib (B-Raf)	Cobimetinib (MEK)	Atezolizumab (PD-L1)	*BRAF* V600 (any variant)	FDA-Approved (2020; IMspire150)	[15]
**Any Advanced Melanoma—Dual Immunotherapy**					
Nivolumab (PD-1)	Ipilimumab (CTLA-4)	—	Any (*BRAF*-mutant or wild-type)	FDA-Approved (2015; CheckMate 067)	[16]

Table 1 summarizes the currently FDA-approved combination treatment regimens for melanoma.

**Table 2:** Selected investigational combination regimens for melanoma (clinical and preclinical).

Drug 1 (Target)	Drug 2 (Target)	Drug 3 (Target)	Mutation Context	Study Type	Ref.
***BRAF*** ** V600-Mutant—Investigational Triplets**					
Encorafenib (B-Raf)	Binimetinib (MEK)	Pembrolizumab (PD-1)	*BRAF* V600E/K	Phase I/II (IMMU-TARGET)	[17]
Encorafenib (B-Raf)	Binimetinib (MEK)	Pembrolizumab (PD-1)	*BRAF* V600E/K	Phase III, ongoing (STARBOARD)	[18]
Dabrafenib (B-Raf)	Trametinib (MEK)	Pembrolizumab (PD-1)	BRAF V600E/K	Phase II (KEYNOTE-022)	[9]
Binimetinib (MEK)	Nivolumab (PD-1)	Ipilimumab (CTLA-4)	*BRAF* V600-mutant (brain mets)	Phase II (SWOG S2000)	[19]
***BRAF*** ** V600-Mutant—MEK + ICI Doublets**					
Trametinib (MEK)	Pembrolizumab (PD-1)	—	*BRAF*-mutant (late-line)	Retrospective/case series	[20]
Cobimetinib (MEK)	Atezolizumab (PD-L1)	—	*BRAF* V600E/K	Phase III (arm within IMspire150)	[15]
***NRAS*** **-Mutant—MEK + CDK4/6 Inhibition**					
Binimetinib (MEK)	Ribociclib (CDK4/6)	—	*NRAS*-mutant	Phase Ib/II	[21]
***NRAS*** **-Mutant—MEK + HDAC Inhibition**					
Binimetinib (MEK)	Bocodepsin (HDAC)	—	*NRAS*-mutant	Phase Ib/2 (Nautilus)	[22]
***NRAS*** **-Mutant—RAF + MEK Inhibition**					
Trametinib (MEK)	Naporafenib (pan-RAF)	—	*NRAS*-mutant	Phase Ib	[23]
Cobimetinib (MEK)	Belvarafenib (RAF dimer)	—	*NRAS*-mutant	Phase Ib	[24]
***NRAS*** **-Mutant—MEK Monotherapy**					
Tunlametinib (MEK)	—	—	*NRAS*-mutant	Phase II (monotherapy)	[25]
**RAS-Mutant Solid Tumors—BCL-xL + MEK Inhibition**					
Trametinib (MEK)	Navitoclax (BCL-xL)	—	*KRAS*- or *NRAS*-mutant (basket)	Phase I/II	[26]
***HRAS*** **-Mutant—Preclinical/Early-Phase**					
ASN007 (ERK1/2)	Copanlisib (PI3K)	—	RAS-mutant (preclinical)	Preclinical	[27]
Tipifarnib (FTI)	—	—	*HRAS*-mutant	Phase II—no clinical benefit	[28]
***MET*** **-Preclinical**					
Tepotinib (c-MET)	—	—	*MET* overexpressed	Preclinical (cell line WM451)	[29]

Table 2 summarizes selected investigational combination therapies currently being evaluated in clinical trials for melanoma.

## Data Availability

This article is a review of previously published literature and does not report original research data. All sources used are cited in the reference list and are publicly available through PubMed.

## Abbreviations

The following abbreviations are used in this manuscript:

AKT	Refers to three serine/threonine kinases: Akt1, Akt2, and Akt3.
AST	Abbreviation of aspartate aminotransferase.
BRAF (protein)	RAF isoform. Shortened name for BRAF proto-oncogene serine/threonine kinase.
BRAF (proto-oncogene)	The gene that encodes for BRAF.
BRAFi	Abbreviation of BRAF inhibitors.
CDK 4/6	Refers to two cyclin-dependent kinases. An abbreviation of cyclin-dependent kinases 4 and 6.
c-Jun	Transcription factor protein. Shortened name for transcription factor Jun.
c-Raf	RAF isoform. Shortened name for RAF proto-oncogene serine/threonine-protein kinase. RAF isoform.
CTLA-4	Abbreviation of cytotoxic T-lymphocyte-associated protein 4.
dMMR	Referring to a tumor cell’s inability to properly repair DNA damage. Abbreviation of deficient mismatch repair.
DOR	Duration of Response.
ECOG PS	Eastern Cooperative Oncology Group Performance Status.
ERK 1/2	Refers to two members of the MAPK family. An abbreviation of extracellular signal-regulated protein kinases 1 and 2.
Fd-7	Receptor protein. Abbreviation of Frizzled-7.
FTI	Abbreviation of farnesyltransferase inhibitors.
GAP	Abbreviation of GTPase-activating protein.
GGT	Abbreviation of gamma-glutamyl transferase.
GGTase-1	Abbreviation of geranylgeranyltranferase-I.
GTPase	An enzyme that binds to guanosine triphosphate and hydrolyzes it, turning the molecule into guanosine diphosphate.
HDACs	A family of enzymes. An abbreviation of histone deacetylases.
HGF	Growth factor protein. Abbreviation of hepatocyte growth factor.
HNSCC	Abbreviation of Head and Neck Squamous Cell Carcinoma.
HR	Abbreviation of hazard ratio.
HRAS	RAS isoform. Shortened form of Harvey Rat sarcoma viral oncogene homolog.
ICIs	Immune checkpoint inhibitors.
IHC	Abbreviation of immunohistochemistry.
JAK2	Refers to both the human gene and the non-receptor tyrosine kinase encoded by the *JAK2* gene. With regard to the protein, JAK2 is an abbreviation of Janus kinase 2.
JNKs	Mitogen-activated protein kinases. Abbreviation of c-Jun terminal kinases.
KRAS	RAS isoform. Shortened form of Kirsten rat sarcoma virus oncogene homolog.
LDH	Lactate dehydrogenase.
MAPK	A type of serine/threonine-specific protein kinase which includes ERKs, JNKs, and p38s. Abbreviation of mitogen-activated protein kinase.
MEK 1/2	Refers to two dual-specificity kinase enzymes. An abbreviation of dual-specificity mitogen-activated protein kinase kinase 1 and 2.
MEKi	Abbreviation of MEK inhibitors.
MSI-H	Referring to a tumor cell’s inability to properly repair DNA replication errors. Abbreviation of microsatellite instability-high.
mTOR	A regulatory serine-threonine protein kinase. Abbreviation of mechanistic target of rapamycin.
NGS	Next-generation sequencing
NRAS	RAS isoform. Shortened form of neuroblastoma Ras viral oncogene homolog.
OS	Overall survival.
PD-1	Programmed cell death protein 1.
PD-L1	Programmed cell death ligand 1.
PI3Ks	A family of kinase enzymes. Abbreviation of Phosphoinositide 3-kinases.
PFS	Progression-free survival.
PTEN	A tumor suppressor gene. Abbreviation of phosphatidylinositol-3,4,5-trisphosphate 3-phosphatase.
RAF	A family of serine/threonine-specific protein kinases. Abbreviation of rapidly accelerated fibrosarcoma.
RTKs	Receptor tyrosine kinases.
RYKs	Atypical member of the RTK family. Abbreviation of receptor-like tyrosine kinases.
TAZ	A transcriptional regulatory protein. Abbreviation of transcriptional coactivator with a PDZ-binding motif.
TKIs	Tyrosine kinase inhibitors.
UV	Abbreviation for ultraviolet.
V600E	*BRAF* gene missense mutation that replaces valine (V) with glutamic acid (E) at amino acid 600.
V600K	*BRAF* gene missense mutation that replaces valine (V) with lysine (K) at amino acid 600.
Wnt5a	Signaling glycoprotein. Abbreviation of wingless type MMTV integration site family member 5A.
YAP	A transcriptional regulatory protein. Abbreviation of yes-associated protein 1.
